# Castor Bean Epidemic

**Published:** 1853-02

**Authors:** 


*Castor Bean Epidemic.*—One of the steamboats running between Cincinnati and St. Louis, on a recent trip, was crowded with German emigrants. As might be expected, their appetite for fruit and vegetables, after a long sea voyage, was most voracious. At Selma, a short distance below St. Louis, the boat received some fifteen or twenty sacks of castor beans. Their appearance excited the cravings of the emigrants. Finally, curiosity and appetite triumphed; a bag was surreptitiously opened, a large panfull extracted, and a huge luncheon of soup prepared. In a short time, the passengers in the cabin and the officers of the boat were startled by the report that the cholera, in its worst form, had broken out on deck: the castor oil was doing its work. The bag lay exposed, and a large potfull was steaming hot on the table. The captain was ordered into quarantine.—*Boston Med. and Surg. Jour.*

